# Characterization of *Bifidobacterium kashiwanohense* that utilizes both milk- and plant-derived oligosaccharides

**DOI:** 10.1080/19490976.2023.2207455

**Published:** 2023-05-15

**Authors:** Kento Orihara, Kana Yahagi, Yuki Saito, Yohei Watanabe, Toshio Sasai, Taeko Hara, Naoki Tsukuda, Kaihei Oki, Junji Fujimoto, Takahiro Matsuki

**Affiliations:** Basic Research Department, Yakult Central Institute, Tokyo, Japan

**Keywords:** *Bifidobacterium kashiwanohense*, xylan, human milk oligosaccharide, plant-derived carbohydrate, extracellular xylanase, ABC transporter SBP

## Abstract

Bifidobacteria are prominent members of the human gut microbiota throughout life. The ability to utilize milk- and plant-derived carbohydrates is important for bifidobacterial colonization of the infant and adult gut. The *Bifidobacterium catenulatum* subspecies *kashiwanohense* (*B. kashiwanohense*) was originally isolated from infant feces. However, only a few strains have been described, and the characteristics of this subspecies have been poorly investigated. Here, we characterized genotypes and phenotypes of 23 *B. kashiwanohense*-associated strains, including 12 newly sequenced isolates. Genome-based analysis clarified the phylogenetic relationship between these strains, revealing that only 13 strains are genuine *B. kashiwanohense*. We defined specific marker sequences and investigated the worldwide prevalence of *B. kashiwanohense* based on metagenome data. This revealed that not only infants but also adults and weaning children harbor this subspecies in the gut. Most *B. kashiwanohense* strains utilize long-chain xylans and possess genes for extracellular xylanase (GH10), arabinofuranosidase and xylosidase (GH43), and ABC transporters that contribute to the utilization of xylan-derived oligosaccharides. We also confirmed that *B. kashiwanohense* strains utilize short- and long-chain human milk oligosaccharides and possess genes for fucosidase (GH95 and GH29) and specific ABC transporter substrate-binding proteins that contribute to the utilization of a wide range of human milk oligosaccharides. Collectively, we found that *B. kashiwanohense* strains utilize both plant- and milk-derived carbohydrates and identified key genetic factors that allow them to assimilate various carbohydrates.

## Introduction

Bifidobacteria are commonly detected as prominent members of the human gut throughout life. Bifidobacterial colonization has been associated with a reduced risk of obesity, ^1^ infection,^2^ and allergy.^3–5^ These bacteria are well-adapted to the gut environment. Recent studies have demonstrated that their ability to utilize microbiota-accessible carbohydrates is a key factor enabling their stable persistence in the human gut.^6–8^

To date, hundreds of bifidobacterial strains have been isolated, and the characteristics of major bifidobacterial species have been described. Most of these studies involved comparative and functional genome analyses.^6,9–12^ Microbiota analysis of infants and adults revealed that the bifidobacterial species colonizing the human gut change with age.^13,14^ Specifically, *Bifidobacterium breve*, *Bifidobacterium bifidum*, and *Bifidobacterium longum* subspecies *infantis* (hereafter, *B. infantis*) are generally predominant in infants, whereas *Bifidobacterium adolescentis*, and *B. pseudocatenulatum* are prevalent in adults. *B. longum* subspecies *longum* (hereafter, *B. longum*) is distributed in both infant and adults.^15^

Recent studies demonstrated that adult-prevalent bifidobacterial species can utilize plant-derived carbohydrates, including xylan-based oligosaccharides (e.g., xylooligosaccharides [XOS] and arabinoxylooligosaccharides [AXOS]), and starch-related carbohydrates (e.g., amylopectin, pullulan, maltotriose, maltodextrin, and their derived oligosaccharides).^8,16,17^ Molecular mechanisms of utilization of these plant-derived carbohydrates have only just started to be elucidated. Based on previous studies, enzymes belonging to glycoside hydrolase (GH) family 43 (GH43) and others (e.g., GH51, GH8, GH120, and GH10) are involved in xylan-related glycan utilization,^8,17^ whereas enzymes belonging to GH13 are associated with starch utilization.^6^ Furthermore, ATP-binding cassette (ABC) transporters, usually encoded in the same operon as these GHs, also play important roles in the uptake of these oligosaccharides.^17^

Homologs of the glycosidase and transporter genes are shared among adult-associated bifidobacteria. However, each adult-associated bifidobacterial species exhibits different glycan preferences. *B. pseudocatenulatum* possesses more abundant GH43 genes and ABC transporters for xylan-based oligosaccharides utilization than other adult-associated bifidobacteria,^8,17^ suggesting that this species assimilates a broader range of xylan-based oligosaccharides than other bifidobacterial species. On the other hand, *B. adolescentis* possesses numerous GH13 genes^6,18^ and accumulates in resistant starch granules in the human gut, suggesting starch preference of this species.^16,19^

By contrast, the infant-associated species do not utilize plant-derived carbohydrates as well as the adult-prevalent species. However, they readily utilize human milk oligosaccharides (HMO).^20^ HMOs are non-digestible oligosaccharides present in breast milk that include various glycan structures. Fucosyllactose (FL, including 2′-FL and 3-FL) is the main component of HMO, and genetic factors responsible for its utilization (i.e., fucosidase and ABC transporters) have been intensively investigated and characterized.^7,20,21^

Inter- and intra-species variation in the utilization of various HMO components by bifidobacterial species and the underlying molecular mechanisms have been reported. *B. infantis* utilizes HMO most efficiently compared with other *Bifidobacterium* species. The subspecies imports various HMOs via a number of ABC transporters, where an intracellular glycosidase subsequently hydrolyzes the imported oligosaccharides into monosaccharides.^22,23^ Consequently, *B. infantis* assimilates the broadest range of HMO components compared with other bifidobacteria; however, the gene maintenance costs involved with equipping various transporters may be high.^24^ *B. bifidum* is the second most efficient HMO metabolizer among bifidobacteria. The species hydrolyzes HMOs using extracellular glycosidases, and the resultant mono- and di-saccharides are then taken up by its limited number of transporters.^25^ The species breaks down most HMOs with lower protein-equipping cost than *B. infantis*; however, the resultant extracellular saccharides can be utilized by other gut microbes, and *B. bifidum* does not assimilate HMO efficiently in competition. In contrast, *B. breve* cannot utilize long-chain HMOs [e.g., lacto-*N*-fucopentaose (LNFP) and lacto-*N*-difucohexaose (LNDFH)], and only a subset of *B. breve* strains can utilize FL.^7,26^ The FL-utilizing *B. breve* strains import FL via the well-characterized ABC transporter,^7,20^ and the imported FL is subsequently hydrolyzed to lactose and fucose by an intracellular GH95 fucosidase. The utilization system is efficient since *B. breve* strains can utilize the main HMOs (i.e., 2′-FL and 3-FL) relying on a limited number of transporters and a glycosidase.

*B. kashiwanohense*, which we are going to characterize in this study, was isolated from Japanese infant feces in 2011.^27^ The species was later reclassified as a subspecies of *B. catenulatum* based on digital DNA – DNA hybridization analysis.^28^ This subspecies has been rarely detected in humans, and only a few strains have been isolated from infant feces. Some studies have reported that *B. kashiwanohense* strains can utilize FL.^11,26,29^ However, the utilization of other carbohydrates, and the global and age-dependent prevalence of this subspecies, are not well understood.

In the current study, we characterized the genotypes and phenotypes of *B. kashiwanohense*. We performed comparative genome analyses of 23 *B. kashiwanohense*-associated strains, including 12 newly sequenced isolates. Based on the phylogenetic analysis, the strains were classified into four groups, and only 13 strains were sensu stricto *B*. *kashiwanohense*. Metagenome data-based investigation of worldwide prevalence revealed that while *B. kashiwanohense* is not often detected in infants and adults, the subspecies is predominant in some weaning children. Most *B. kashiwanohense* strains can utilize long-chain xylans and possess an extracellular xylanase, as well as ABC transporters and intercellular GHs known to contribute to the utilization of xylan-based oligosaccharides. We also confirmed that *B. kashiwanohense* can utilize short- and long-chain HMOs and investigated the underlying molecular mechanisms.

## Results

### *Phylogenic relationship between* B. kashiwanohense-*associated strains*

We assessed the phylogenetic relationship among 23 *B. kashiwanohense*-associated strains by analyzing their genome sequences (Table 1). Of these, 12 strains were sequenced within the current study and 11 were retrieved from the National Center for Biotechnology Information (NCBI) database. The analyzed strains included well-characterized *B. kashiwanohense* strains (JCM 15,439^T^ and APCKJ1),^26,27^ 7 strains of *Bifidobacterium catenulatum* subspecies *catenulatum* (thereafter, *B. catenulatum*), and 3 strains that have previously been identified as *B. kashiwanohense*.^30,31^

**Table 1.** *Bifidobacterium kashiwanohense* associated strain list^26,27,30–37.^

Strain	Isolation	Contig	Genome Size (Mbp)	No. of CDSs	Accession Number	Reference
*B. kashiwanohense* JCM 15,439^T^	Japanese infant feces	1	2.34	1,956	GCA_001042615	Morita et al.(2015)
*B. kashiwanohense* APCKJ1	Irish infant feces	1	2.45	2,099	GCA_009684555	James et al. (2019)
*B. kashiwanohense* YIT 13,051	Japanese child feces	28	2.35	2,024	JAOPLX000000000	This study
*B. kashiwanohense* YIT 13,052	Japanese child feces	16	2.33	1,962	JAOPLY000000000	This study
*B. kashiwanohense* YIT 13,053	Japanese child feces	15	2.34	1,975	JAOPLZ000000000	This study
*B. kashiwanohense* YIT 13,054	Japanese child feces	23	2.26	1,898	JAOPMA000000000	This study
*B. kashiwanohense* YIT 13,055	Japanese child feces	19	2.37	1,989	JAOPMB000000000	This study
*B. kashiwanohense* YIT 13,056	Japanese child feces	18	2.28	1,904	JAOPMC000000000	This study
*B. kashiwanohense* YIT 13,057	Japanese infant feces	56	2.52	2,155	JAOPMD000000000	This study
*B. kashiwanohense* YIT 13,058	Japanese infant feces	30	2.43	2,131	JAOPME000000000	This study
*B. kashiwanohense* YIT 13,060	Belgian infant feces	31	2.37	2,015	JAOPMF000000000	This study
*B. kashiwanohense* YIT 13,061	Belgian infant feces	17	2.3	1,958	JAOPMG000000000	This study
*B. kashiwanohense* YIT 13,062	Belgian infant feces	32	2.53	2,249	JAOPMH000000000	This study
*B. catenulatum* JCM 1194^T^	Human feces	1	2.08	1,702	GCA_001025195	Morita et al. (2015)
*B. catenulatum* 1899B	Infant feces	14	2.12	1,758	GCA_002075855	Duranti et al. (2017)
*B. catenulatum* BIOML-A1	Human feces	18	2.06	1,737	GCA_009160805	Poyet et al. (2019)
*B. catenulatum* YIT 13,059	Japanese infant feces	19	2.03	1,622	JAOPMI000000000	This study
[*B. kashiwanohense*] PV20–2	Kenyan infant feces	1	2.37	1,991	GCA_000800455	Vazquez-Gutierrez et al. (2015)
[*B. kashiwanohense*] N4G05	Kenyan vaginal fluid	12	2.07	1,678	GCF_002742445	Freitas & Hill (2017)
[*B. kashiwanohense*] N5G01	Kenyan vaginal fluid	8	2.12	1,714	GCF_002742425	Freitas & Hill (2017)
[*B. catenulatum*] GCF_901212515	Bangladeshi children feces	28	2.23	1,842	GCA_901212515	Cowardin et al. (2019)
[*B. catenulatum*] GCF_902167655	Bangladeshi children feces	36	2.17	1,802	GCA_902167655	Raman et al. (2019)
[*B. catenulatum*] GCF_902167905	Bangladeshi children feces	75	2.18	1,853	GCA_902167905	Gehrig et al. (2019)

**Figure 1.**
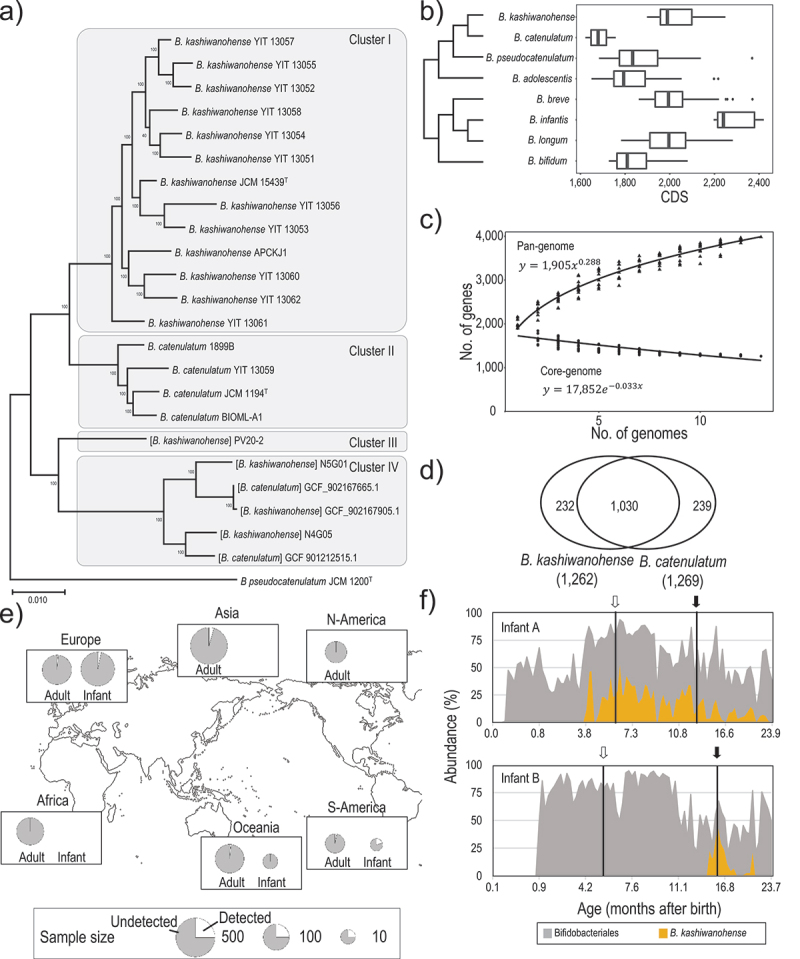
General genome features of *B. kashiwanohense*.

The phylogenetic tree, constructed based on the single-nucleotide polymorphisms (SNPs) of 776 core genes of the 23 strains, divided the strains into four major clusters (Figure 1a). Cluster I included the *B*. kashiwanohense type strain JCM 15,439^T^ and 11 newly sequenced strains. Cluster II included *B. catenulatum* strains (JCM 1194^T^, 1899B, YIT 13,059, and BIOML-A1). Strain PV20–2, reported as *B. kashiwanohense* by Vazquez-Gutierrez et al.,^31^ was not included in cluster I nor II. The average nucleotide identity (ANI) values of PV20–2 against the strains in cluster I (*B. kashiwanohense*) and II (*B. catenulatum*) were approximately 95% (Fig. S1), suggesting that PV20–2 should be reclassified as a new subspecies of *B. catenulatum* (designated as cluster III). The strains N5G01 and N4G05, reported as *B. kashiwanohense* by Freitas and Hill,^30^ and 3 putative *B. catenulatum* strains isolated from Bangladeshi children formed 1 cluster (cluster IV, Figure 1a). Since the ANI values among these 5 strains exceeded 95%, and the ANI values against the cluster I, II, and III strains were below 95% (Fig. S1), these 5 strains are likely a new species. Based on these analyses, we proceeded to characterize the 13 strains in cluster I as *B. kashiwanohense*.

(a) Phylogenic tree of *B. kashiwanohense*-associated strains based on their core genome single nucleotide polymorphisms (SNPs). (b) Number of coding sequences (CDSs) in human bifidobacterial species. (c) Pan-genome and core-genome of *B. kashiwanohense*. (d) Venn diagram of core genes of *B. kashiwanohense* and *B. catenulatum*. (e) Distribution of *B. kashiwanohense* on each continent. The size of the pie chart represents the number of metagenomes used for estimating the distribution of this subspecies. (f) Abundance of the Bifidobacteriales, including *B. kashiwanohense*, during the first 2 years of life^14^. White and black arrows represent the initiation of solid food and cessation of breastfeeding, respectively.

### *General genome features of* B. kashiwanohense

Genome-based analysis revealed the median number of protein-coding sequences (CDS) of 1989, which is higher than those of the closely related bifidobacterial species (i.e., *B. catenulatum* and *B. pseudocatenulatum*) (Figure 1b). The pan-genome and core genome harbored 3,983 and 1,262 genes, respectively (Figure 1c). Comparison of the core genomes of *B. kashiwanohense* and *B. catenulatum* revealed that 1,030 genes were shared between the subspecies, while 232 and 239 genes were unique to *B. kashiwanohense* and *B. catenulatum*, respectively (Figure 1d).

Next, we examined the worldwide distribution of *B. kashiwanohense* using metagenome data deposited in public databases (Supplementary data 2, 3). We used two complete genome sequences to define the *B. kashiwanohense*-specific sequence (K-mer) using the bioinformatic tools Kraken2^38^ and Bracken^39^ (see Materials and Methods for details). We attempted to detect *B. kashiwanohense*-specific sequences in 1,193 sets of metagenomic data (data for 289 infants from 6 countries and 904 adults from 14 countries) deposited in the NCBI database (Figure 1e and Table S1). We detected *B. kashiwanohense* sequences not only in infants but also in adults. However, the number of *B. kashiwanohense*-positive subjects was limited: only 23 subjects among 904 adults (2.54%) and 11 subjects among 289 infants (3.81%) harbored the bacteria in the gut. We did not observe any differences in the prevalence of this subspecies in different continents. Additionally, we examined the *B. kashiwanohense* distribution in two metagenome-assembled genomes (MAG) database^40,41^. Compared with other Bifidobacteria, fewer *B. kashiwanohense* MAGs were detected, which supported our result from metagenomic data showing the limited distribution of this subspecies (Supplementary data 4).

Previously, we engaged in research on infant gut microbiota development using dense longitudinal sampling.^14^ In the present study, we detected *B. kashiwanohense* in 3 out of 12 infants during their first 2 years of life (data for 2 infants are shown in Figure 1f). Previous studies reported that bifidobacterial species composition changes after weaning based on the carbohydrate utilization phenotype (e.g., infant-associated bifidobacteria efficiently utilize milk oligosaccharides, whereas adult-associated bifidobacteria utilize dietary fiber-derived oligosaccharides). However, *B. kashiwanohense* maintained gut colonization before and after cessation of breastfeeding. This observation is consistent with the carbohydrate utilization phenotype of this subspecies, as discussed in the following subsections.

### Bifidobacterial genotypes and phenotypes associated with carbohydrate utilization

**Figure 2.**
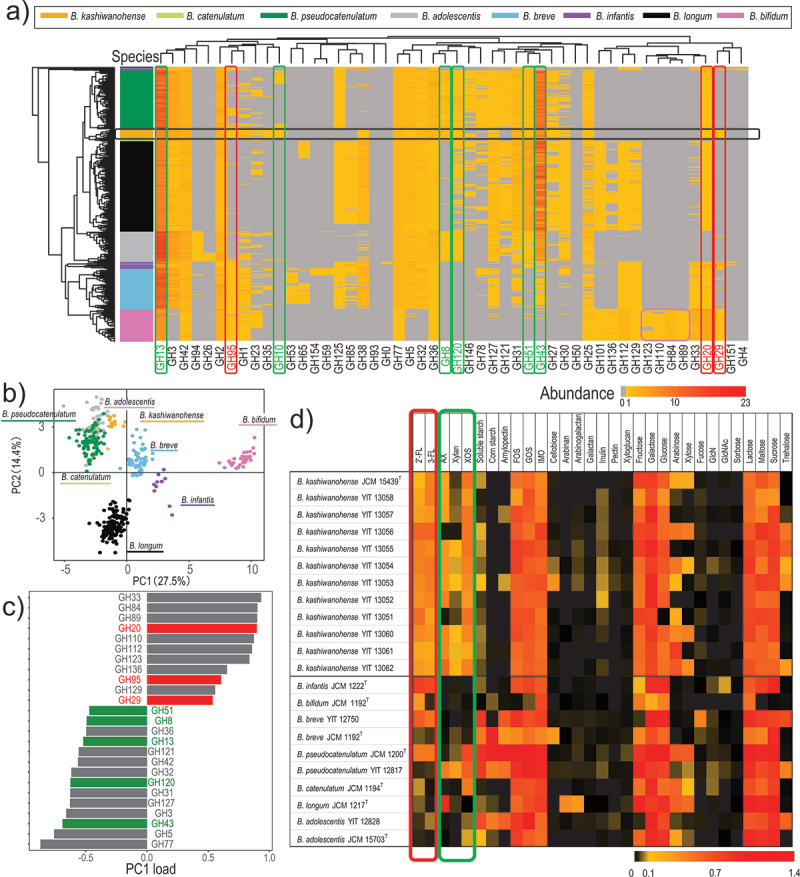
*B. kashiwanohense* glycobiome and associated growth profiles.

Note: (a) Glycosyl hydrolase (GH) family gene profiles of 13 *B. kashiwanohense* strains in comparison with other bifidobacterial strains (n = 350 genomes in total). The strains (y-axis) and GH family scale abundance (x-axis) have been hierarchically clustered by measuring the Euclidean distance with complete linkage clustering. A black box in the figure represents the GH profiles of *B. kashiwanohense*. The red and green boxes represent the HMO utilization and plant-derived carbohydrates utilization GH genes, respectively, that are harbored by *B. kashiwanohense* strains. The pink box represents *B. bifidum* specific GH genes. (b) Principal component analysis (PCA) of the GH profile of each strain. (c) Explained variance plot. Red and green represent featured GH genes associated with HMO and plant-derived carbohydrate metabolism, respectively. (d) Growth of *B. kashiwanohense* and other *Bifidobacterium* strains with different carbohydrates as substrates. The growth of each strain was determined as optical density (OD_600_) at the end of cultivation (60 h), and the result is visualized in the heatmap.

To better understand the carbohydrate utilization ability of *B. kashiwanohense*, we compared the abundance of carbohydrate-active enzymes (CAZy) in the subspecies with those of other human-associated bifidobacteria. We visualized the GH gene profile of each strain using a heatmap (Figure 2a) (Supplementary data 5). Hierarchal clustering based on the scaled abundance of GH genes confirmed that the GH profile is largely species-dependent (Figure 2a, left). Principal component analysis (PCA) of the GH profiles revealed that infant-associated species (e.g., *B. infantis*, *B. breve*, and *B. bifidum*) and adult-associated species (*B. adolescentis*, *B. catenulatum*, and *B. pseudocatenulatum*) are separated along the PC1 axis, and *B. longum* was plotted at the middle of infant- and adult-association species (Figure 2b).

The top 25 principal component loads associated with PC1 are shown in Figure 2c and Table S2. *B. bifidum* exhibited a unique GH profile (i.e., GH84, GH89, GH110, and GH123: pink box in Figure 2a). The infant-associated species were characterized by the presence of genes for GHs known to contribute to HMO utilization (e.g., GH20, GH29, and GH95, Figure 2c: Table S2, top), while the adult-associated species were characterized by genes for enzymes associated with plant-derived carbohydrate utilization (e.g., GH8, GH10, GH13, GH43, GH51, and GH120) (Figure 2c: Table S2, bottom).

In the analysis, we also found that *B. kashiwanohense* strains were plotted between infant- and adult-associated species (Figure 2b). Consistent with previous studies, these strains harbor GH genes involved in HMO utilization (e.g., GH20, GH29 and GH95: red box in Figure 2a). Of note, most strains belonging to this subspecies also possess abundant genes for glycosyl hydrolases for xylan, starch, and their-derived oligosaccharide utilization (e.g., GH8, GH10, GH13, GH43, GH51, and GH120: green box in Figure 2a).

We next investigated the growth of *B. kashiwanohense* strains in the presence of 32 different carbohydrates as the sole carbon source, including plant- and host-derived glycans (i.e., HMOs and mucin), in comparison with that of strains of bifidobacterial species commonly found in the human gut (Figure 2d). We confirmed that most infant-associated bifidobacteria (i.e., *B. infantis*, *B. bifidum*, and some *B. breve* strains), all *B. kashiwanohense* strains, and some *B. pseudocatenulatum* strains utilize the major HMOs (i.e., 2′-FL and 3-FL) (Figure 2d), while most adult-associated bifidobacteria (i.e., *B. adolescentis* and some *B*. *pseudocatenulatum* strains) are unable to utilize these oligosaccharides (i.e., they exhibited limited growth in a medium containing these molecules). Furthermore, we confirmed that most infant-associated species (*B. infantis* and *B. bifidum*) did not efficiently utilize plant-derived carbohydrates (e.g., xylan, XOS, and starch), whereas *B. kashiwanohense* and adult-associated bifidobacteria utilized these carbohydrates (green box in Figure 2d).

Based on the *in silico* genome analysis (Figure 2a–c) and *in vitro* growth experiments (Figure 2d), we next moved to link specific genes with the observed carbohydrate utilization phenotype.

### B. kashiwanohense*strains can utilize the dietary fiber arabinoxylan*

**Figure 3.**
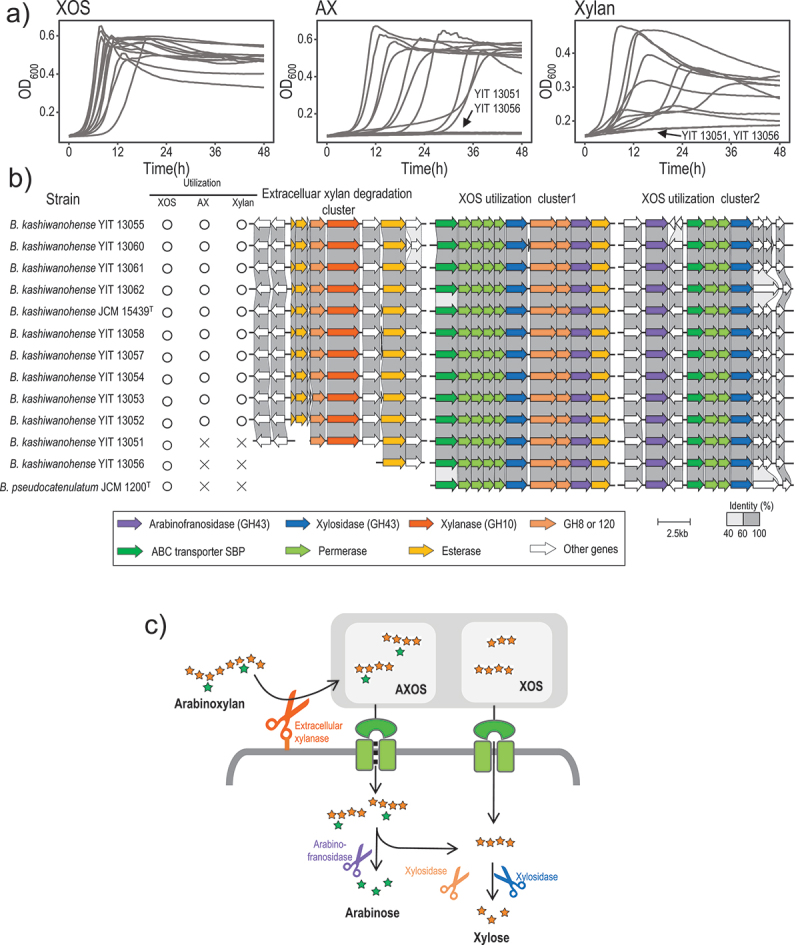
Utilization of xylan-associated carbohydrates by *B. kashiwanohense*.

Note: (a) Growth curves of 12 *B. kashiwanohense* strains cultured with xylooligosaccharide (XOS), arabinoxylan (AX), and xylan as substrates. (b) Organization of genes for xylan-related carbohydrate utilization (right) and the associated phenotype (left). The shadows (grayscale) represent the homology among strains. (c) Proposed metabolic pathway of AX conversion to monosaccharides. The glycosidases and transporters involved in xylan-related carbohydrate utilization are colored as in Figure 3b.

The utilization of plant-derived carbohydrates by *B. kashiwanohense* subspecies has not been investigated to date. In the current study, we found that most *B. kashiwanohense* strains could utilize arabinoxylan, xylan, and their derived oligosaccharides (Figure 3a). Previous studies^17^ demonstrated that adult-associated bifidobacteria, such as *B. pseudocatenulatum*, can utilize xylan-based oligosaccharides, and possess genes for ABC transporters and GHs (e.g., GH43, GH51, GH8, and GH120). These transporters and GHs work together to utilize xylan-based oligosaccharides of different sizes and with different side residue modiﬁcations. In the current study, we found that all *B. kashiwanohense* strains possess homologs of the *B. pseudocatenulatum* XOS utilization genes involved in XOS uptake and hydrolysis, most of which are encoded in XOS utilization clusters 1 and 2 (Figure 3b).

Recently, we found that some *B. pseudocatenulatum* strains possess an extracellular xylanase gene from the GH10 family enzyme, which plays an important role in the utilization of long-chain xylans.^8^ In the current study, we observed that most xylan-utilizing *B. kashiwanohense* strains encode the xylanase homolog, while the xylan-non-utilizing strain YIT 13,056 does not (Figure 3b). This suggests the importance of GH10 ×ylanase in long-chain xylan utilization by *B. kashiwanohense* (Figure 3c). The xylan-non-utilizing strain YIT 13,051 harbors the xylanase gene, however, the operon that includes the xylanase gene is incomplete, which might be associated with its inability to utilize xylans (Figure 3b).

### B. kashiwanohense*-specific ABC transporter contributes to short- and long-chain HMO utilization*

**Figure 4.**
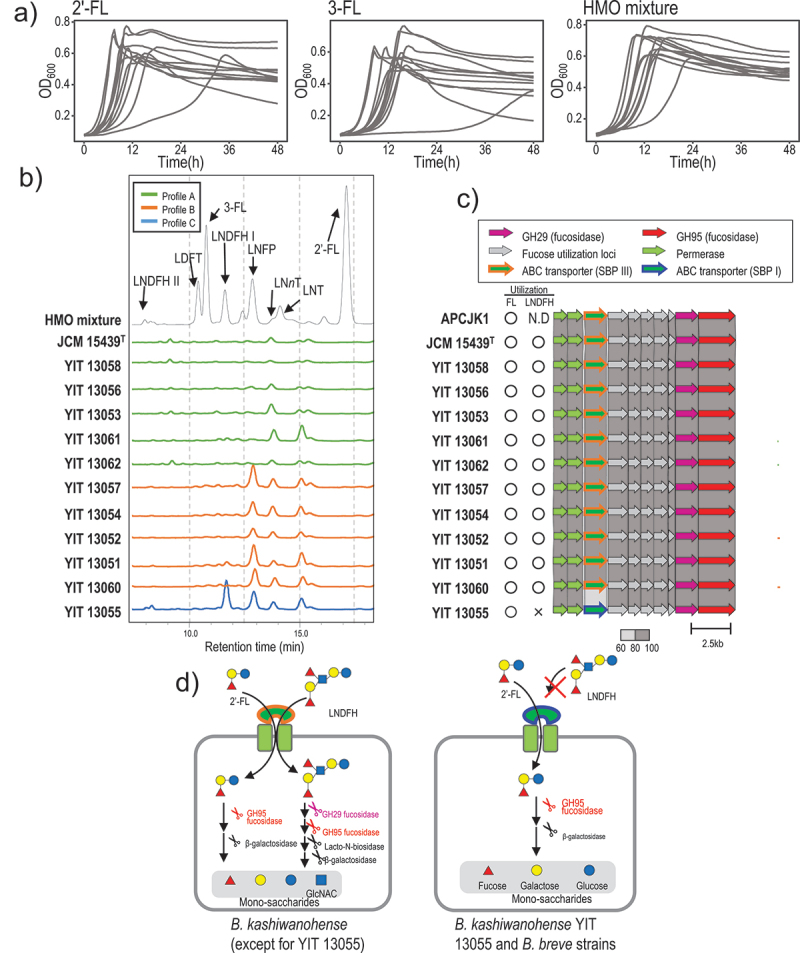
HMO utilization by *B. kashiwanohense*.

Note: (a) Growth curves of 12 *B. kashiwanohense* strains cultured in the presence of 2′-FL, 3-FL, and HMO mixture. (b) HPLC profiles of the HMOs remaining in the culture supernatants after 72 h. (c) Organization of genes for HMO utilization and the associated phenotype. A difference in ABC transporter SBP subtypes between LNDFH-utilizing and non-utilizing strains was noted. (d) Model of HMO utilization by *B. kashiwanohense* strains. The glycosidases and transporters involved in HMO utilization are colored as in Figure 4d.

In agreement with previous studies,^26,29^ we observed that all *B. kashiwanohense* strains utilize FL (i.e., 2′- FL and 3-FL, Figure 4a). We evaluated the ability of *B. kashiwanohense* to utilize other HMOs using purified breast milk oligosaccharides. We cultivated *B. kashiwanohense* strains in a medium containing an HMO mixture for 72 hours (Figure 4a, right) and then used high-performance liquid chromatography (HPLC) to investigate the oligosaccharides remaining in the culture supernatant (Figure 4b, Figure S2). Based on HPLC analysis, we observed three major HMO utilization profiles: 6 out of 12 strains utilized most HMOs (profile A, shown in green in Figure 4b), 5 strains did not utilize any LNFP structural isomers (profile B, shown in orange in Figure 4b), whereas the remaining strain (YIT 13,055) did not utilize LNFP as well as LNDFH I (profile C, shown in blue in Figure 4b).

Previous studies have demonstrated the importance of GHs and the ABC transporter substrate-binding protein (SBP) in HMO utilization.^7,22^ Therefore, we next attempted to associate HMO utilization phenotypes with the presence of GH and SBP genes. We performed orthologous gene clustering analysis using the bioinformatics tool Roary^42^ (see Materials and Method for more details). The results are shown in Fig. S3. We searched for a gene whose presence corresponded with the LNFP and LNDFH utilization phenotype. No GH genes were associated with the LNFP and LNDFH utilization phenotypes (Fig. S3a, b), and no SBP genes were associated with the LNFP utilization phenotype (Fig. S3a, c). However, the presence of one SBP gene (ID1343) corresponded with the LNDFH-utilization phenotype (Fig. S3a, c, red box).

ID1343 is a homolog of SBP that is essential for FL utilization.^7,22^ According to previous studies, there are several SBP subtypes for FL (type I – IV).^7,22,43^ Of note, SBP associated with LNDFH utilization represented a unique subtype (type III) that was only present in *B. kashiwanohense* strains (Fig. S4). In addition, we observed that the LNDFH-non-utilizing strain YIT 13,055 harbors a gene for a FL-SBP that belongs to another subtype (type I) (Figure 4c and Fig. S4). In other words, the differences in LNDFH utilization observed in *B. kashiwanohense* strains correspond to those in their FL-SBP subtype (Figure 4c). The result suggests that the subtype of ABC transporter SBP specific to *B. kashiwanohense* (type III) is involved not only in FL but also long-chain HMO utilization (Figure 4d). This is in good agreement with a recent publication by Ojima et al., showing that *B. kashiwanohense* specific FL-SBP (type III) mediates the uptake of long chain HMO, based on the phylogenetic analysis of bifidobacterial SBP.^43^ Our gene-trait matching approach additionally showed this gene is well conserved among *B. kashiwanohense* strains.

## Discussion

**Figure 5.**
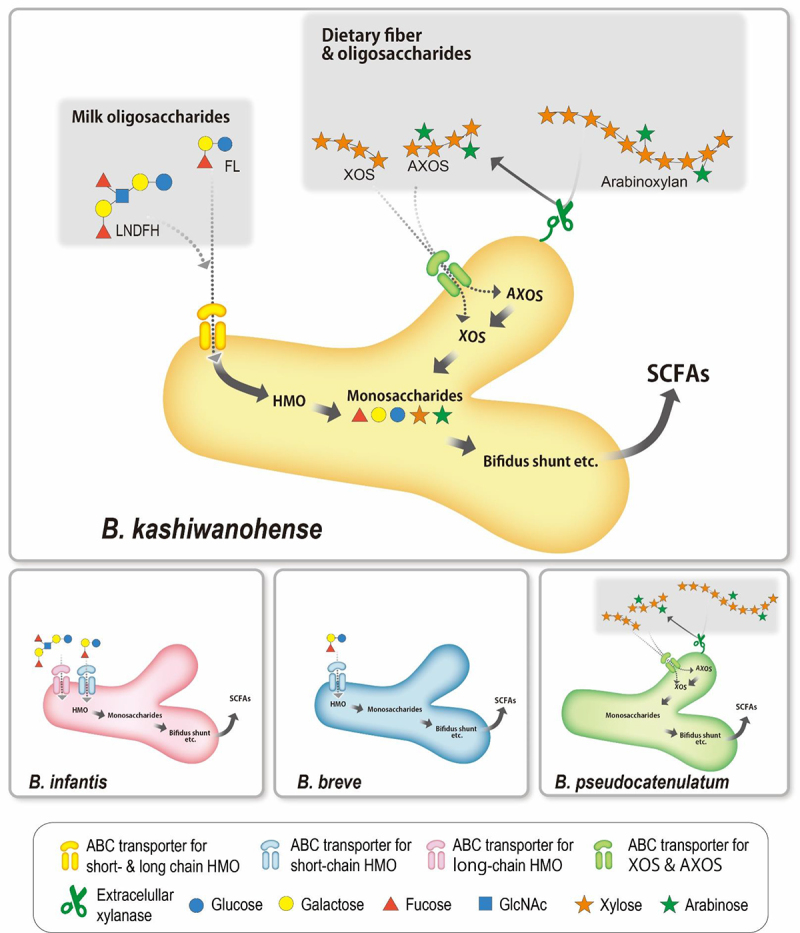
*B. kashiwanohense* strains utilize both milk- and plant-derived carbohydrates.

Note: *B. kashiwanohense* strains possess unique SBP that transports not only short- but also long-chain HMOs. Most *B. kashiwanohense* strains possess an extracellular xylanase homolog that suggests it enables primary degradation of arabinoxylan, ABC transporters for xylan-based oligosaccharides, and an intercellular xylosidase. This carbohydrate utilizing strategy is unique and contrasts that of infant-associated bifidobacteria (e.g., *B. infantis* and *B. breve*) utilizing HMOs and adult-associated species (e.g., *B. pseudocatenulatum*) utilizing dietary fiber.

In the current study, we characterized the genotypes and phenotypes of *B. kashiwanohense*. We found that this subspecies can utilize not only short- and long-chain HMOs but also dietary fiber arabinoxylan and its derived oligosaccharides. By using the gene – trait matching approach, we found the unique metabolic pathways involved in and the key genetic factors for utilizing this wide range of substrates (Figure 5).

The utilization of plant-derived carbohydrates has not been investigated in this subspecies; in the current study, we revealed that most *B. kashiwanohense* strains can utilize xylans and their derived oligosaccharides (Figure 3a). Previous studies reported that some human gut bacteria belonging to Bacteroidetes (synonym Bacteroidota) and Firmicutes (synonym Bacillota) possess an extracellular xylanase (i.e., xylanolytic activity) and play central roles in the primary degradation of xylans,^44,45^ while bifidobacterial species, such as *B. adolescentis* and *B. longum*, have long been recognized to take advantage of xylan-derived oligosaccharides produced by primary degraders via substrate cross-feeding.^46–48^ Although long-chain xylan utilization by some *B. pseudocatenulatum* strains has been reported recently,^8^ primary degradation of xylan is rare among bifidobacterial strains. In the current study, we found that most *B. kashiwanohense* strains encode an extracellular xylanase, which enables them to act as primary degraders of xylan.

Since *B. kashiwanohense* strains can utilize a wide range of microbiota-accessible carbohydrates (e.g., HMOs and plant-derived carbohydrates), we assumed that *B. kashiwanohense* may have a competitive advantage in the complex environment of the human gut. However, our metagenome analysis of *B. kashiwanohense* distribution suggested that the prevalence of this subspecies is limited in both infants and adults. This suggests that carbohydrate utilization constitutes just one aspect of the competitive advantage in the complex microbial environment, and other factors exist that underpin wide bacterial distribution and predominance in the human gut.

Our analysis revealed that *B. kashiwanohense* strains are detectable and predominant in the gut before and after weaning, indicating that *B. kashiwanohense* may have evolved to adapt to the gut environment during the weaning period. This concept is in good agreement with a recent study,^49^ showing that a clade of *B. longum* possessing both HMO- and dietary-fiber-utilizing-genes expanded during the weaning period. It will be interesting to evaluate the association between bifidobacterial carbohydrate utilization expansion during the weaning by using in vitro competitive experiments and/or assessed using future infant cohorts, to support the concept.

In the current study, we found that *B. kashiwanohense* strains can utilize not only FL but also long-chain HMOs, including LNFP and LNDFH, using the gene – trait matching analysis. These findings are in good agreement with a recent publication by Ojima et al.^43^ who analyzed the differences in substrate specificity for FL among SBPs. The authors reported that the *B. kashiwanohense*-specific SBP contributes to the transportation of FL, as well as LNDFH and LNFP I/II, but not LNFP III.^43^ In the current study, we found little remaining LNFP in the culture supernatant of some *B. kashiwanohense* strains with specific FL-SBP (type III) (Figure 4c, Fig. S2). This result suggested that these strains consumed not only LNFP I/II with the SBP for FL but also LNFP III through another uptake system (Fig. S5). Although we did not identify the putative SBP for LNFP III, the accumulation of the *B. kashiwanohense* genome and HMO utilization phenotypes observed in this study may contribute to future investigations.

## Conclusion

In this study, we conducted comparative genome analysis for 23 *B. kashiwanohense*-associated strains (including 12 new isolates), worldwide (metagenomics) prevalence analysis, and their carbohydrate utilization to characterize the subspecies. The genotype and phenotype analyses revealed that the subspecies can utilize plant-derived carbohydrates and short- and long-chain HMOs. Our detailed characterization of *B. kashiwanohense* suggests that each bifidobacterial species employs different and unique strategies to colonize the human gut and may contribute to our understanding of the reason for the life-long predominance of bifidobacteria (and the change in species with age).

## Materials and methods

### Bacterial strains and culture

The strains used in the current study were obtained from the Japan Collection of Microorganisms (JCM; Ibaraki, Japan) and Yakult Culture Collection (YIT; Tokyo, Japan). The strains were routinely cultured at 37°C in an anaerobic chamber (Coy Laboratory, Grass Lake, MI, USA) with 88% N_2_, 5% CO_2_, and 7% H_2_, using mGAM broth (Nissui Pharma, cat. 05422) containing 0.5 w/vol% glucose and 0.5 w/vol% lactose. Evaluation of *Bifidobacterium* carbohydrate utilization profiles was performed at 37°C in modified ILS-PIPES (100 mM PIPES, pH 7.1, 5 g/L yeast extract, 10 g/L trypticase peptone, 3 g/L tryptose, 1 mL/L Tween 80, 0.3 g/L l-cysteine hydrochloride, 575 mg/L MgSO_4_･7 H_2_O, 154.5 mg/L MnSO_4_･5 H_2_O, 34 mg/L FeSO_4_･7 H_2_O, and 2 g/L diammonium hydrogen citrate) supplemented with the targeted carbohydrates (0.5 w/vol%). Growth curves were evaluated by measuring the OD_600_ every 30 min using a microplate reader PowerWave 340 (BioTek, Winooski, VT, USA) in an anaerobic chamber.

### HMO glycoprofiling

The HMO mixture was prepared as described previously.^7^ Bacteria were grown in a medium containing HMO mixtures for 72 h. According to our previous report,^7^ the oligosaccharides remaining in the culture supernatant at the end of the experiment were labeled with *p*-aminobenzoic acid ethyl ester as an ultraviolet light-absorbing compound and quantified using HPLC. The labeled HMO components were separated using a Shimadzu Prominence HPLC system (Kyoto, Japan) with an L-column 2 ODS (Chemicals Evaluation and Research Institute, Tokyo, Japan). Modified HMOs were eluted with a 13:87 (vol:vol) mixture of acetonitrile and 100 mM ammonium acetate (pH 4.5) at 40°C and were detected using an SPD-20 A ultraviolet detector (Shimadzu) at 304 nm. The following oligosaccharides were used as controls: 2′-FL (Advanced Protein Technologies), 3-FL (Dextra Laboratories, cat L303), LNFP I (Dextra Laboratories, cat L502), LNDFH I (Dextra Laboratories, cat L602), LNDFH II (Dextra Laboratories, cat L603), LNT (IsoSep AB, cat 45/01–0010), lacto-*N*-neotetraose (IsoSep AB, cat 45/08–0010), DFL (IsoSep AB, cat 45/02–0010), LNFP II (IsoSep AB, cat 55/06–0001), LNFP III (IsoSep AB, cat 55/07–0005), and *N*-acetyl glucosamine (Nacalai Tesque, cat 00520–16).

### Genome sequencing

As described previously, DNA was extracted from bifidobacterial strains using a bead – phenol method.^8^ A DNA library was prepared using the TrueSeq DNA PCR-Free Library Preparation Kit (Illumina, San Diego, CA, USA) and sequenced on MiSeq (Illumina) using the MiSeq Reagent kit V2 (250 bp × 2) (Illumina). The output paired-end reads were assembled into contigs by Unicycler (v0.4.8)^50^ or A5-miseq (v20160825),^51^ which were then aligned to the complete genome of *B. kashiwanohense* JCM 15,439^T^ (accession ID: GCA_001042615.1) using Mauve (2015_02_13).^52,53^

### Genome analysis

The ANIb values between strains (Fig. S1) were calculated using pyani (v0.2.9).^54^ For genomic feature comparison among bifidobacterial species (listed in Supplementary data 1), the CDSs number was calculated using Prokka (v1.14.6).^55^ The number of pan-genome and core-genome genes was evaluated using Roary (v3.13.0)^42^ using default settings. A phylogenetic tree of *B. kashiwanohense-*associated strains (Figure 1a) was constructed based on SNPs from core-genome gene alignment using FastTree (v2.1.10).^56^ To evaluate the diversity within the species, the numbers of unique and common genes in the core-genome genes of two sub-species, *B. kashiwanohense* and *B. catenulatum*, were determined using Roary^42^ with the type strain for each sub-species. Synteny of genes for carbohydrate utilization (i.e., xylan-associated carbohydrates and HMOs utilization) was visualized using clinker^57^ with default settings.

### B. kashiwanohense*distribution analysis using public metagenome data and MAG databases*

Kraken2 (v2.0.9 beta)^38,58^ and Bracken (v2.6.0)^39^ were used to investigate the global distribution of *B. kashiwanohense* from shotgun metagenomic data (listed in Supplementary data 1, 2). *B. kashiwanohense-*specific K-mers were defined using *B. kashiwanohense* JCM 15,439 and APCKJ1. To avoid false positives, the confidence score and the relative abundance cutoff value were carefully adjusted; the confidence score was set to 0.9 and the relative abundance cutoff value to 0.49%, so that the false-positive and false-negative rate for the evaluation dataset^14^ were 4.9% and 8.2%, respectively. High quality Bifidobacterial MAGs were retrieved from previous reports.^40,41^ The MAGs were filtered by contamination (≤5%), completeness (≥90%), number of contigs (≤500), and contig N50 (≥10kb). The *B. kashiwanohense* MAGs were defined by ANIb value (≥97%) to the *B. kashiwanohense* type strain (JCM 15,439^T^) genome.

### GH gene profiling and visualization

Genes in the pan-genome of all bifidobacterial strains (*n* = 350, Figure 2a) were annotated for the CAZy database^59^ using dbCAN2.^60,61^ The CDSs were considered CAZy genes only if they were annotated using HMMER, DIAMOND, and Hotpep pipeline with default parameters. To normalize the count data of CAZy genes, the “scale” function in R (v4.0.5) was used. To visualize the GH profiles in a heat map format (Figure 2a), the “heatmap.2” function in R package “gplot” was used. PCA was performed using the “prcomp” function with default settings in R. The raw data including CBM is shown in Supplementary data 5. In Figure 3b, genes considered as esterase were annotated by Prokka^55^, BlastKoala,^62,63^ or dbCAN2.

## Supplementary Material

Supplemental Material

Supplemental Material

## Data Availability

The bifidobacterial genome sequences have been deposited in the NCBI Sequence Read Archive under BioProject Accession Code PRJNA883016 (https://www.ncbi.nlm.nih.gov/bioproject/PRJNA883016)
