# Supplementary material for: Characterization of Bifidobacterium kashiwanohense that utilizes both milk- and plant-derived oligosaccharides
Source: Gut Microbes. 2023 May 15;15(1):2207455. doi: 10.1080/19490976.2023.2207455 (PMC10187079; doi:10.1080/19490976.2023.2207455)
Supplement: Supplemental Material [file KGMI_A_2207455_SM5457.pdf]

	JCM 15439^{T}	APCKJ1	YIT 13057	YIT 13058	YIT 13062	YIT 13061	YIT 13060	YIT 13051	YIT 13052	YIT 13053	YIT 13054	YIT 13055	YIT 13056	JCM 1194^{T}	1899B	BIOML-A1	YIT 13059	PV20-2	GCF_902167905.1	GCF_902167655.1	GCF_901212515.1	N4G05	N5G01
JCM 15439^{T}	100	98.3	98.6	98.5	98.2	98.4	98.2	98.4	98.5	98.8	98.6	98.4	98.2	96.6	96.7	96.4	96.0	95.3	93.5	93.5	94.3	94.4	94.0
APCKJ1	98.3	100	98.2	98.2	98.1	98.1	98.3	97.9	98.1	97.8	98.3	97.9	97.5	96.4	96.6	96.2	96.0	95.4	93.8	93.7	94.3	94.3	94.0
YIT 13057	98.8	98.3	100	99.1	98.1	98.0	98.1	98.6	99.1	98.5	99.0	99.1	97.8	96.2	96.4	96.1	95.8	95.2	93.7	93.6	94.3	94.3	94.0
YIT 13058	98.5	98.1	99.0	100	98.0	98.0	98.1	98.6	98.9	98.3	98.9	98.5	97.8	96.3	96.5	96.1	95.9	95.2	93.5	93.5	94.2	94.2	93.9
YIT 13062	98.2	98.0	98.0	98.0	100	98.1	98.5	97.7	98.0	97.7	98.1	97.8	97.3	96.5	96.6	96.3	96.0	95.4	93.7	93.7	94.5	94.4	94.1
YIT 13061	98.4	98.0	97.9	98.0	98.1	100	98.2	97.9	98.1	98.1	98.2	97.8	97.6	96.8	96.7	96.5	96.2	95.3	93.8	93.8	94.3	94.4	94.0
YIT 13060	98.2	98.3	98.1	98.1	98.6	98.2	100	97.9	98.1	97.8	98.3	98.0	97.2	96.5	96.4	96.4	96.1	95.4	93.7	93.7	94.3	94.2	94.0
YIT 13051	98.4	97.8	98.5	98.5	97.7	97.9	97.9	100	98.8	98.4	98.9	98.4	97.9	96.3	96.5	96.2	96.0	95.1	93.4	93.4	94.2	94.1	94.0
YIT 13052	98.5	98.2	99.1	98.9	98.0	98.0	98.2	98.9	100	98.7	99.0	99.1	98.0	96.2	96.4	96.1	95.8	95.2	93.5	93.5	94.2	94.2	94.0
YIT 13053	98.8	97.9	98.4	98.3	97.8	98.1	97.8	98.4	98.6	100	98.6	98.4	98.8	96.2	96.3	95.9	95.6	95.0	93.3	93.3	94.0	94.1	93.8
YIT 13054	98.6	98.3	98.9	98.8	98.2	98.2	98.3	98.8	99.0	98.6	100	98.8	97.9	96.2	96.4	96.2	95.8	95.3	93.5	93.5	94.3	94.4	94.0
YIT 13055	98.5	98.0	99.1	98.6	97.9	97.9	98.0	98.4	99.1	98.4	98.8	100	97.8	96.0	96.0	95.9	95.5	95.0	93.4	93.4	94.0	94.0	93.8
YIT 13056	98.3	97.5	97.8	97.8	97.4	97.6	97.2	97.9	98.0	98.7	97.9	97.8	100	95.9	95.9	95.6	95.7	94.8	93.3	93.3	93.9	94.0	93.8
JCM 1194^{T}	96.5	96.4	96.2	96.2	96.4	96.6	96.4	96.2	96.2	96.0	96.2	95.8	95.8	100	98.2	98.5	98.3	94.9	93.7	93.8	93.7	93.8	93.7
1899B	96.7	96.5	96.3	96.5	96.6	96.6	96.4	96.5	96.5	96.2	96.5	96.0	95.9	98.3	100	98.3	98.0	94.8	93.5	93.5	93.8	93.9	93.8
BIOML-A1	96.3	96.3	96.2	96.1	96.2	96.5	96.4	96.3	96.2	95.9	96.2	95.9	95.6	98.6	98.3	100	98.2	95.1	93.8	93.8	93.9	93.9	93.7
YIT 13059	96.0	96.0	95.7	95.8	96.0	96.1	96.0	95.9	95.8	95.5	95.8	95.4	95.5	98.3	97.9	98.2	100	94.8	93.6	93.6	93.7	93.6	93.8
PV20-2	95.3	95.3	95.0	95.2	95.4	95.3	95.3	95.1	95.2	95.0	95.3	95.0	94.7	95.0	94.7	95.0	94.6	100	94.0	94.2	94.8	94.7	94.3
GCF_902167905.1	93.5	93.7	93.5	93.5	93.6	93.8	93.7	93.4	93.5	93.3	93.4	93.3	93.2	93.9	93.4	93.8	93.7	94.1	100	99.9	96.7	96.7	97.9
GCF_902167655.1	93.5	93.7	93.6	93.5	93.6	93.8	93.7	93.5	93.6	93.4	93.5	93.4	93.3	93.9	93.4	93.8	93.8	94.1	99.9	100	96.8	96.7	98.0
GCF_901212515.1	94.2	94.3	94.1	94.2	94.5	94.2	94.2	94.1	94.1	93.9	94.3	94.0	93.8	93.9	93.7	93.9	93.8	94.8	96.8	96.8	100	98.2	97.2
N4G05	94.3	94.3	94.2	94.2	94.5	94.3	94.3	94.1	94.2	94.0	94.4	94.0	94.0	93.9	93.8	93.8	93.6	94.7	96.7	96.8	98.3	100	97.0
N5G01	94.1	94.1	94.0	93.8	94.1	94.1	94.1	94.1	94.0	93.9	94.0	93.9	93.9	93.9	93.8	93.7	93.9	94.4	98.0	98.1	97.3	97.1	100

100 95 93.1

Figure S1. ANI value matrix of *B. kashiwanohense*-related strains. The average nucleotide identity (ANI) values were calculated by pyani.[54]

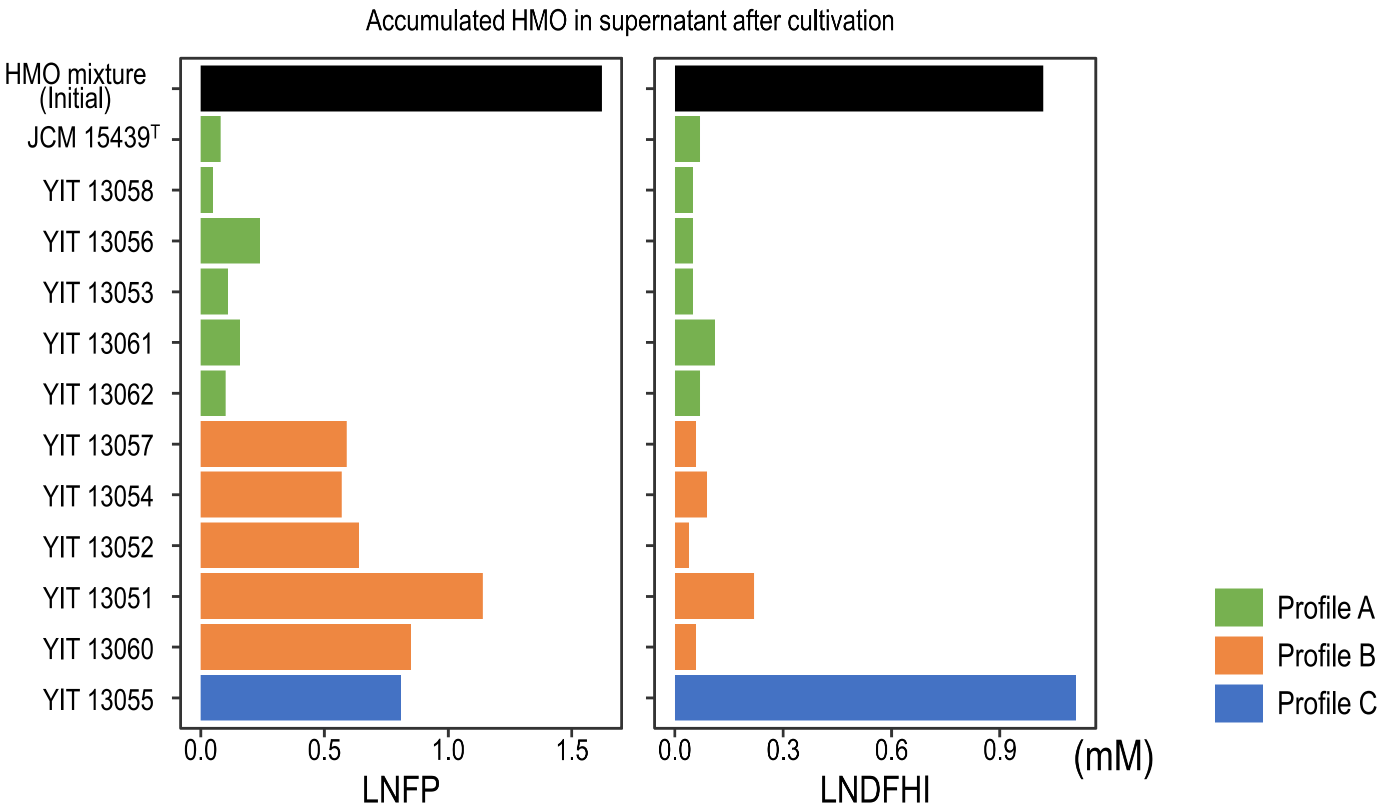

Figure. S2. LNDFH I and LNFP utilization by *B. kashiwanohense*. *B. kashiwanohense* strains were cultured with a mixture of human milk oligosaccharide (HMO) for 72 h. Oligosaccharides remaining in the culture supernatant were then analyzed by HPLC (Fig. 4b). The value means the average of more than 2 experiments (raw data is shown in Supplementary data 6).

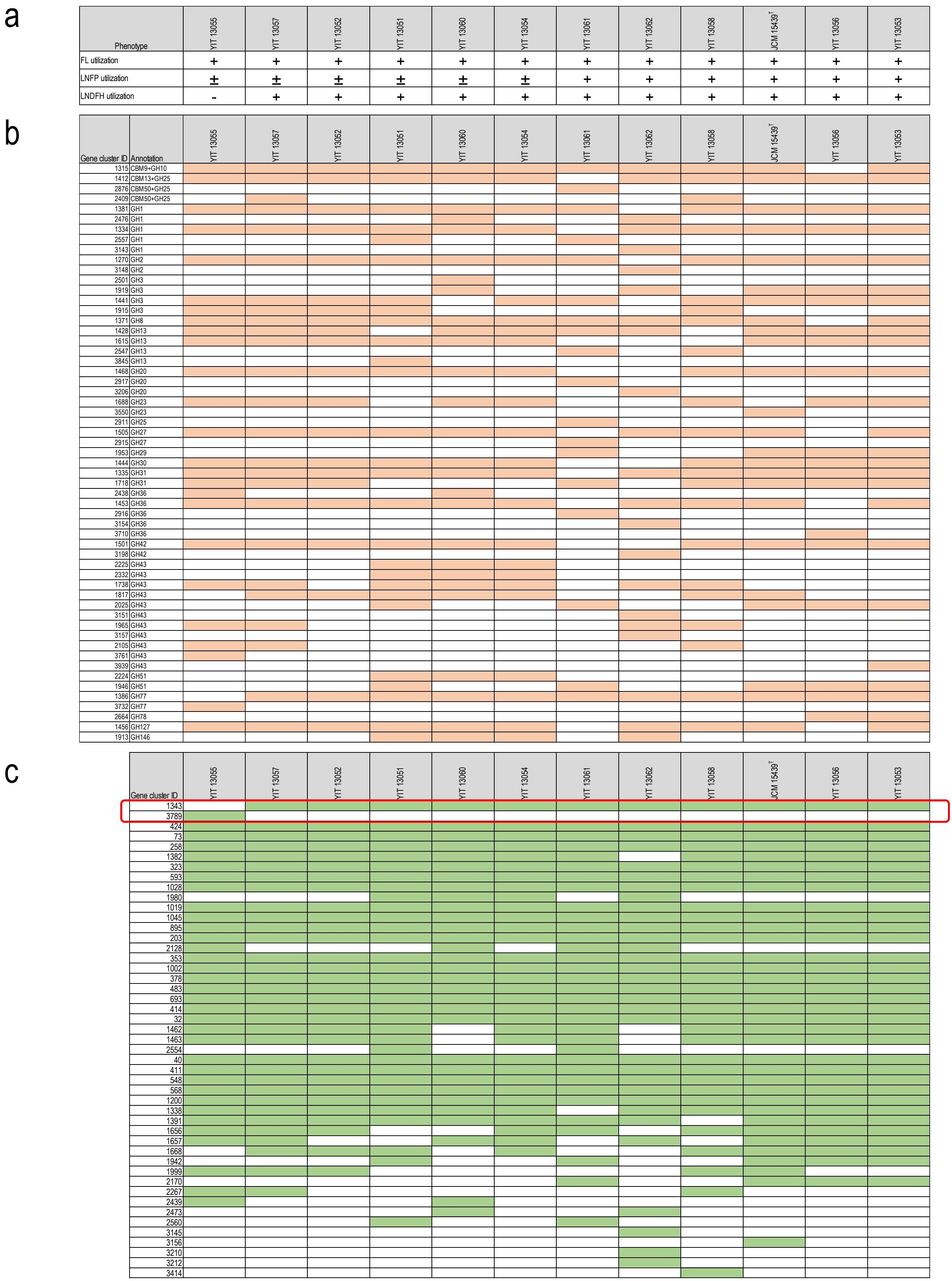

Figure. S3. HMO utilization and the presence of GH and SBP genes. Of 3,983 pan-genome genes, 87 were annotated as GH genes and 47 as ABC transporter SBP genes. (a) The FL, LNFP, and LNDFH utilization ability of all *B. kashiwanohense* strains tested. (b) The presence (light orange) or absence (white) of GH genes in *B. kashiwanohense* strains. GH genes harbored by all *B. kashiwanohense* strains have been omitted from the figure. (c) The presence (light green) or absence (white) of the ABC transporter SBP gene in *B. kashiwanohense* strains. The red box represents the gene clusters corresponding with the LNDFH utilization phenotype.

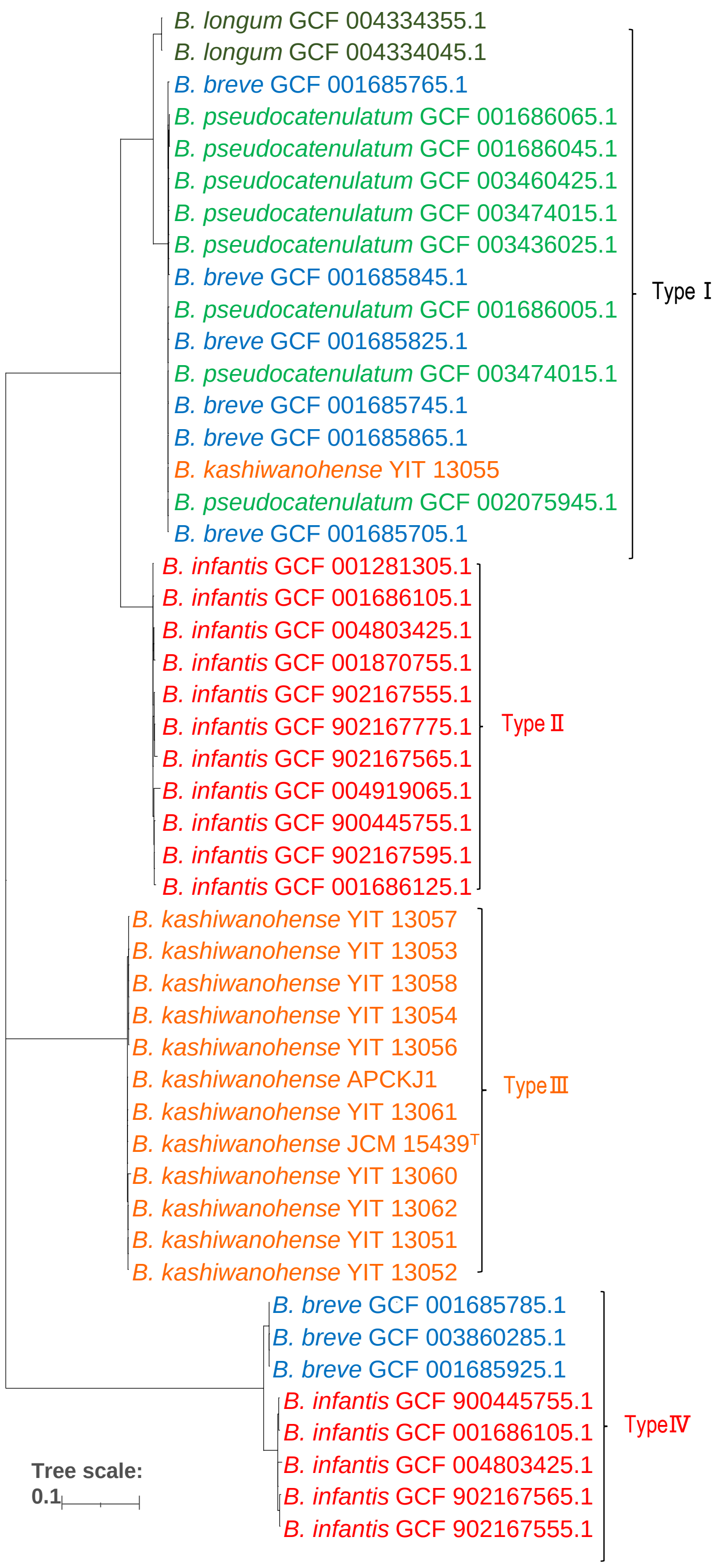

Figure. S4. Clustering of ABC transporter SBP genes for FL into four subgroups. The amino acid sequences of ABC transporter SBP for FL[7] were used as a database to search for homologs in bifidobacterial genomes. BLASTp analysis was performed with an E-value threshold of 10^{-5}. The results were filtered by identity (>50%) and query coverage (>80%). The sequences were aligned using MUSCLE, and the phylogenic tree was created using the maximum-likelihood method with default parameters in MEGAX.[64] The harboring species have been color-coded.

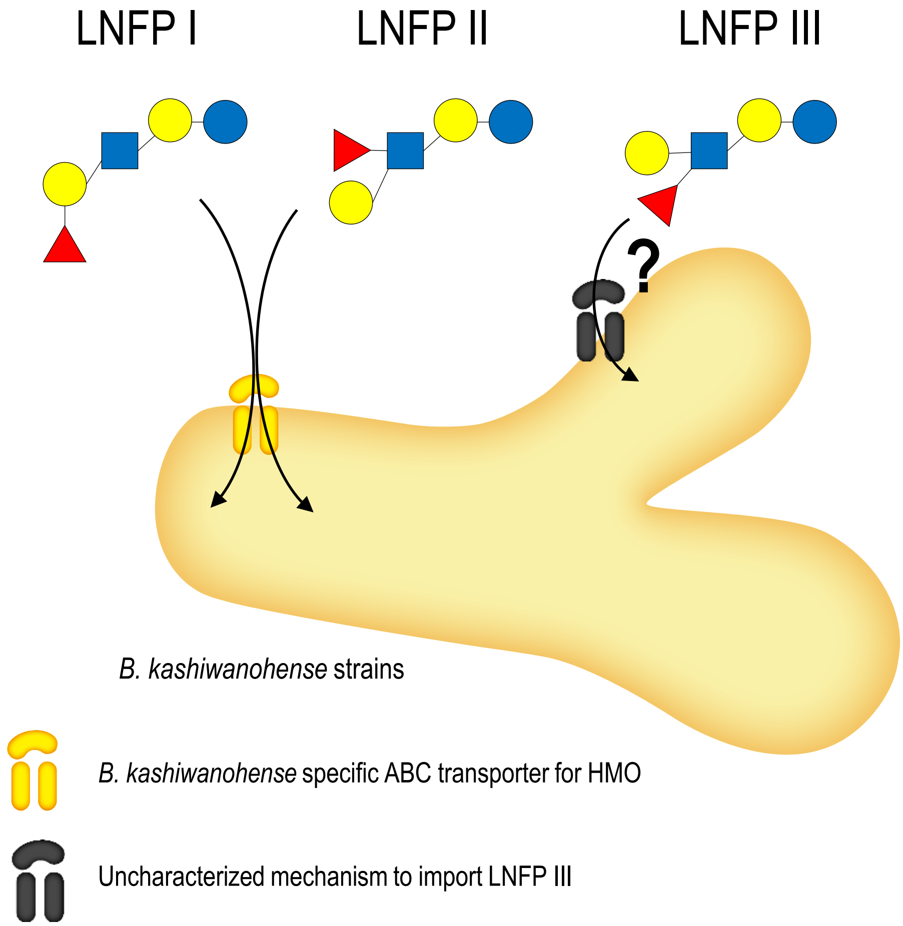

Figure. S5 LNFP transport pathway in *B. kashiwanohense*. LNFP I and II are imported by *B. kashiwanohense* specific ABC transporters.[43] LNFP III may be imported by an uncharacterized mechanism.

Table S1. Global distribution of bifidobacterial species in adults and infants

Age	Continent	Country	sample size	No. of detected subjects						
				B. kashiwa-nohense	*B.catenulatum*	*B. longum*	*B. breve*	*B. bifidum*	*B. pseudo-catenulatum*	*B. adolescentis*
Adults	Africa	Madagascar	95	n. d	n. d	16	n. d	5	9	16
		Tanzania	25	n. d	n. d	n. d	n. d	n. d	n. d	n. d
	Europe	Italy	11	2	2	10	n. d	3	1	9
		Sweden	100	n. d	7	71	3	22	14	61
		Austria	2	n. d	1	2	n. d	1	2	2
		Germany	29	n. d	2	23	n. d	8	6	10
		France	20	n. d	1	10	n. d	4	3	9
	Asia	Kazakhstan	67	1	1	31	2	10	17	40
		Japan	223	17	3	150	24	50	104	103
		China	109	n. d	n. d	11	n. d	2	12	6
	Oceania	Fiji	147	2	n. d	40	2	6	25	85
	N-America	Canada	24	n. d	n. d	7	n. d	n. d	1	3
		USA	22	n. d	n. d	16	1	6	5	7
	S-America	Peru	30	1	n. d	1	1	n. d	5	9
Infants	Europe	Sweden	100	6	6	84	48	36	18	25
		Russia	67	4	8	53	15	30	18	17
		Estonia	57	n. d	2	41	5	17	5	13
		Finland	49	n. d	n. d	35	6	10	9	9
	Oceania	Fiji	11	n. d	n. d	2	n. d	n. d	4	6
	S-America	Peru	5	1	n. d	1	1	1	2	1

n.d., not detected. The subjects were classified as adults (4–59 years old) and infants (0–3 years old). False positives and true positives were identified by adjusting the relative abundance threshold (see Materials and Methods for details).

Table S2 Principal component load associated with PC1.

	Activities in Family	PC1	PC2	PC3	PC4	PC5
GH33	sialidase, etc.	0.25	0.03	0.03	0.05	−0.01
GH84	N-acetyl β-glucosaminidase , etc	0.24	0.05	−0.14	−0.03	0.05
GH89	α-N-acetylglucosaminidase	0.24	0.04	−0.14	−0.03	0.05
GH20	lacto-N-biosidase, β-1,6-N-acetylglucosaminidase, etc	0.24	−0.01	−0.11	0.16	0.02
GH110	α-galactosidase ; α-1,3-galactosidase	0.24	0.05	−0.14	−0.03	0.05
GH112	lacto-N-biose phosphorylase or galacto-N-biose phosphorylase, etc	0.23	−0.16	0.03	−0.05	0.06
GH123	β-N-acetylgalactosaminidase etc	0.23	0.04	−0.13	−0.03	0.05
GH136	lacto-N-biosidase	0.18	−0.10	−0.15	−0.09	0.02
GH95	α-L-fucosidase etc	0.16	0.13	0.19	0.15	0.04
GH129	α-N-acetylgalactosaminidase etc	0.15	−0.23	0.17	−0.04	0.04
GH29	α-L-fucosidase	0.14	0.04	0.04	0.25	−0.33
GH101	endo-α-N-acetylgalactosaminidase	0.10	−0.29	−0.15	−0.11	0.03
GH1	β-glucosidase etc	0.10	0.19	0.16	0.00	0.10
GH23	lysozyme type G etc	0.06	0.07	−0.04	0.06	0.08
GH151	α-L-fucosidase	0.03	−0.02	0.10	0.25	−0.45
GH53	endo-β-1,4-galactanase .	0.03	0.05	0.32	−0.01	0.20
GH4	α-glucosidase etc	0.02	−0.01	0.08	0.19	−0.35
GH0		0.01	−0.01	0.08	0.12	−0.24
GH2	β-galactosidase etc	0.01	0.25	−0.02	0.03	0.22
GH154	β-glucuronidase etc	0.01	0.01	0.21	0.02	0.28
GH93	exo-α-L-1,5-arabinanase	0.01	0.00	0.04	0.00	0.00
GH59	β-galactosidase etc	0.00	0.02	0.20	0.00	0.27
GH65	α,α-trehalase etc	0.00	−0.06	0.19	−0.02	0.16
GH50	β-agarase	−0.01	−0.04	−0.01	−0.04	0.00
GH35	β-galactosidase etc	−0.02	0.11	0.12	0.03	0.07
GH26	β-mannanase etc	−0.03	0.10	0.00	−0.24	−0.14
GH85	endo-β-N-acetylglucosaminidase	−0.03	−0.29	0.14	0.03	−0.05
GH38	α-mannosidase	−0.03	−0.24	0.26	0.01	−0.01
GH25	lysozyme	−0.04	0.03	−0.12	−0.12	−0.03
GH10	endo-1,4-β-xylanase etc	−0.06	0.13	−0.10	0.26	0.06
GH94	cellobiose phosphorylase etc	−0.06	0.17	0.02	−0.41	−0.21
GH78	α-L-rhamnosidase etc	−0.10	0.04	−0.06	0.30	0.25
GH146	β-L-arabinofuranosidase	−0.10	−0.04	−0.16	0.22	0.13
GH125	exo-α-1,6-mannosidase	−0.11	−0.17	0.14	0.29	0.11
GH27	α-galactosidase etc	−0.12	−0.14	−0.18	0.14	0.08
GH30	endo-β-1,4-xylanase etc	−0.13	−0.14	−0.03	−0.24	0.02
GH51	endoglucanase etc	−0.13	−0.27	−0.15	−0.01	−0.01
GH8	endo-1,4-β-xylanase etc	−0.13	0.24	−0.17	0.17	0.01
GH36	α-galactosidase etc	−0.13	0.22	−0.15	0.05	−0.02
GH13	α-amylase etc	−0.14	0.20	0.10	−0.17	−0.01
GH121	β-L-arabinobiosidase	−0.15	−0.12	−0.21	0.08	0.08
GH42	α-L-arabinopyranosidase etc	−0.15	0.20	−0.05	−0.07	−0.10
GH32	invertase etc	−0.17	0.05	0.18	0.03	0.02
GH120	β-xylosidase	−0.17	0.09	−0.19	0.06	−0.02
GH31	α-glucosidase etc	−0.17	−0.16	−0.07	0.04	0.05
GH127	β-L-arabinofuranosidase etc	−0.17	−0.16	−0.04	−0.21	0.04
GH3	β-glucosidase etc	−0.18	0.22	0.10	−0.04	0.02
GH43	β-xylosidase etc	−0.19	−0.12	−0.21	0.04	0.05
GH5	endo-β-1,4-glucanase / cellulase ; endo-β-1,4-xylanase	−0.20	−0.07	0.17	0.06	−0.10
GH77	amylomaltase or 4-α-glucanotransferase	−0.24	−0.04	0.13	0.00	−0.06

Glycosyl hydrolase (GH) family genes are listed in the order of decreasing value of the principal component load of PC1.

Contents of Supplementary data

S-data 1. Genome_data_list
S-data 2. Metagenome_data_list_1 [40, 65-75]
S-data 3. Metagenome_data_list_2
S-data 4. MAG_data [40, 41]
S-data 5. CAZy_gene_raw_data
S-data 6. HMO_profile_raw_data

References in supplementary materials

64. Kumar S, Stecher G, Li M, Knyaz C, Tamura KMX, Battistuzzi FU. Molecular evolutionary genetics analysis across computing platforms. Mol Biol Evol. 2018;35:1547–1549. doi:10.1093/molbev/msy096.
65. Obregon-Tito AJ, Tito RY, Metcalf J, Sankaranarayanan K, Clemente JC, Ursell LK, Zech Xu Z, Van Treuren W, Knight R, Gaffney PM, et al. Subsistence strategies in traditional societies distinguish gut microbiomes. Nat Commun. 2015;6:6505. doi:10.1038/ncomms7505.
66. Qin N, Yang F, Li A, Prifti E, Chen Y, Shao L, Guo J, Le Chatelier E, Yao J, Wu L, et al. Alterations of the human gut microbiome in liver cirrhosis. Nature. 2014;513:59–64. doi:10.1038/nature13568.
67. Nishijima S, Suda W, Oshima K, Kim S-W, Hirose Y, Morita H, Hattori M. The gut microbiome of healthy Japanese and its microbial and functional uniqueness. DNA Res. 2016;23:125–133. doi:10.1093/dnares/dsw002.
68. Yachida S, Mizutani S, Shiroma H, Shiba S, Nakajima T, Sakamoto T, Watanabe H, Masuda K, Nishimoto Y, Kubo M, et al. Metagenomic and metabolomic analyses reveal distinct stage-specific phenotypes of the gut microbiota in colorectal cancer. Nat Med. 2019;25:968–976. doi:10.1038/s41591-019-0458-7.
69. Costea PI, Coelho LP, Sunagawa S, Munch R, Huerta-Cepas J, Forslund K, Hildebrand F, Kushugulova A, Zeller G, Bork P. Subspecies in the global human gut microbiome. Mol Syst Biol. 2017;13:960. doi:10.15252/msb.20177589.
70. Zeller G, Tap J, Voigt AY, Sunagawa S, Kultima JR, Costea PI, Amiot A, Böhm J, Brunetti F, Habermann N, et al. Potential of fecal microbiota for early-stage detection of colorectal cancer. Mol Syst Biol. 2014;10:766. doi:10.15252/msb.20145645.
71. Bäckhed F, Roswall J, Peng Y, Feng Q, Jia H, Kovatcheva-Datchary P, Li Y, Xia Y, Xie H, Zhong H, et al. Dynamics and stabilization of the human gut microbiome during the first year of life. Cell Host & Microbe. 2015;17:690–703. doi:10.1016/j.chom.2015.04.004.
72. Feng Q, Liang S, Jia H, Stadlmayr A, Tang L, Lan Z, Zhang D, Xia H, Xu X, Jie Z, et al. Gut microbiome development along the colorectal adenoma–carcinoma sequence. Nat Commun. 2015;6:6528. doi:10.1038/ncomms7528.
73. Raymond F, Ouameur AA, Déraspe M, Iqbal N, Gingras H, Dridi B, Leprohon P, Plante P-L, Giroux R, Bérubé È, et al. The initial state of the human gut microbiome determines its reshaping by antibiotics. Isme J. 2016;10:707–720. doi:10.1038/ismej.2015.148.
74. Rampelli S, Schnorr SL, Consolandi C, Turroni S, Severgnini M, Peano C, Brigidi P, Crittenden AN, Henry AG, Candela M. Metagenome sequencing of the hadza hunter-gatherer gut microbiota. Curr Biol. 2015;25:1682–1693. doi:10.1016/j.cub.2015.04.055.
75. Vatanen T, Kostic AD, d'Hennezel E, Siljander H, Franzosa EA, Yassour M, Kolde R, Vlamakis H, Arthur TD, Hämäläinen A-M, et al. Variation in microbiome LPS immunogenicity contributes to autoimmunity in humans. Cell. 2016;165:842–853. doi:10.1016/j.cell.2016.04.007.
